# Excess Weight and Dyslipidemia in Seri (Comcáac) Indigenous Children: A Cross-Sectional Study of Prevalences and Associated Factors

**DOI:** 10.3390/epidemiologia7030084

**Published:** 2026-06-16

**Authors:** Yazmín Hugues Ayala, María A. Leal-Serna, Yamili Rojo-Medina, José M. Moreno-Abril, Ana C. Gallegos-Aguilar, Heliodoro Alemán-Mateo, Silvia Y. Moya-Camarena, Araceli Serna-Gutiérrez, Karely Pérez-Gil, Julián Esparza-Romero

**Affiliations:** 1 Obesity and Diabetes Research Unit, Nutrition Coordination, Research Center in Food and Development (CIAD), Gustavo Enrique Astiazarán Rosas No. 46, Col. La Victoria, Hermosillo 83304, Sonora, Mexico; yhugues222@estudiantes.ciad.mx (Y.H.A.); mleal122@estudiantes.ciad.mx (M.A.L.-S.); drojo123@estudiantes.ciad.mx (Y.R.-M.); jmoreno225@estudiantes.ciad.mx (J.M.M.-A.); acristina@ciad.mx (A.C.G.-A.); helio@ciad.mx (H.A.-M.); 2 Molecular Nutrition Laboratory, Nutrition Coordination, Research Center in Food and Development (CIAD), Gustavo Enrique Astiazarán Rosas No. 46, Col. La Victoria, Hermosillo 83304, Sonora, Mexico; moyas@ciad.mx; 3 Sociocultural Department, Sonora Institute of Technology, Cd. Obregon 85137, Sonora, Mexico; aserna@itson.edu.mx; 4 Department of Health Sciences, Cajeme Campus, University of Sonora, Blvd. Bordo Nuevo s/n, Former Ejido Providencia, Cd. Obregón 85010, Sonora, Mexico

**Keywords:** body weight, childhood obesity, native children, lipid screening

## Abstract

**Background/Objectives**: Excess weight and dyslipidemia are health conditions growing worldwide in children, including indigenous populations. The concern is their related comorbidities, which could appear at an early age. Given limited information on Seri children, this study aimed to evaluate the prevalence of excess weight and dyslipidemia, and to identify factors associated with BMI-for-age Z-score and dyslipidemia. **Methods**: This cross-sectional study was conducted among Seri children aged 3–11 years. For prevalence analysis, the BMI-for-age Z-score was calculated as an indicator of excess weight. Dyslipidemia was assessed only in school-age children. Information was collected on potential associated variables, including maternal nutritional status, children’s sleep behaviors, physical activity, diet, and cardiovascular health, as well as household characteristics such as the modernity index and food insecurity. **Results**: A total of 154 Seri children were evaluated. Among preschoolers, 18.8% were classified at risk of overweight. In school-age children, the combined prevalence of overweight and obesity was 32.8%. Maternal BMI and weight, the modernity index, and being a boy were positively associated with the BMI-for-age Z-score, whereas having food insecurity, cardiovascular health score, and sleep time were negatively associated. Dyslipidemia prevalence was 46.1% among school-age Seri children. Having dyslipidemia was positively associated with maternal BMI, percentage of energy intake from ultra-processed products, paternal occupation as a merchant, and child age, and negatively associated with the number of remunerative maternal economic activities. **Conclusions**: These findings provide evidence on the prevalence of excess weight and dyslipidemia and their associated factors among Seri children and may inform future research and health strategies in Seri and other vulnerable populations.

## 1. Introduction

Obesity is characterized by excessive adiposity accumulation, which may occur with or without abnormalities in the distribution or function of adipose tissue [1]. The comorbidities of excess weight were once considered health problems only in adulthood; nowadays, children are developing chronic non-communicable diseases such as cardiovascular disease, hypertension, and type 2 diabetes [2]. Overweight in children can also cause short-term adverse health outcomes, including altered blood lipids, immune, gastrointestinal, respiratory, and musculoskeletal disorders, as well as lower self-esteem, poorer academic performance, and an increased risk of obesity in adulthood [2,3]. Dyslipidemia is a metabolic complication commonly associated with obesity, characterized by abnormal lipid levels in the blood [4]. It is an early marker of cardiovascular risk and can persist into adult years if left untreated, increasing the risk of insulin resistance, endothelial dysfunction, and atherosclerosis [4,5]. In children, dyslipidemia may remain undetectable, making early screening essential in high-risk populations, especially in those with a high prevalence of obesity [4].

Excess weight was traditionally considered a problem primarily in high-income countries; however, it is now prevalent in low- and middle-income populations worldwide, affecting all age groups [2,6]. In Mexico, 7.5% of children under five years and 36.6% of school-aged children (5–11 years) have overweight or obesity [7,8]. In the state of Sonora, slightly higher rates have been reported, with 10.1% among children under 5 years and 38.2% among school-aged children [7,8]. Indigenous school-age children in Mexico also show high prevalences of overweight and obesity, including Maya (50.9%), Otomi (28.4%), Mixtec (36.5%), and Yaqui children (35.6%) [9,10].

There are no national data on the prevalence of childhood dyslipidemia in Mexico; however, two studies have reported this condition in Mexican indigenous populations. Among Yaqui children aged 6–9 years, a prevalence of 38.6% was reported [11]. Another study, including children aged 5–13 years from urban and rural communities and from Yaqui and Seri indigenous groups, found prevalences of 52.0% in Yaqui and 46.3% in Seri children [12]. Differences across studies should be interpreted with caution, as there is no consensus on the classification of dyslipidemia in children, and different cut-off points were used.

In addition to documenting the prevalence of excess weight and dyslipidemia, an essential step in research is to understand the associated factors of BMI-for-age Z-score (used to define excess weight in children) and dyslipidemia. While a hypercaloric diet and low physical activity are commonly considered as major drivers of obesity, they account for only a portion of the factors that contribute to weight gain in children. Indeed, evidence suggests that the development of excess weight in Mexican children is a complex interplay of individual characteristics and economic, social, and family health factors [7,8]. Unfortunately, the evidence on Mexican indigenous children is limited. In school-aged Yaqui children from Sonora, maternal body mass index (BMI), birth weight, screen time, and energy intake were positively associated with BMI-for-age Z-score; in contrast, food insecurity was negatively associated [9].

By contrast, the factors associated with dyslipidemia in Mexican children have been studied far less extensively. Among Yaqui children, a higher BMI-for-age Z-score, increased waist circumference, a family history of diabetes, and paternal permanent employment have been identified as risk factors. In contrast, a higher physical activity score appeared to be protective against dyslipidemia among children in this indigenous group [11]. However, evidence on the association between excess weight and dyslipidemia in other Mexican indigenous children remains scarce, highlighting the need to study populations with diverse cultural and environmental contexts.

The Seri people are a small indigenous population in northern Mexico, living along the coast of the Sonoran Desert, with distinctive cultural and environmental characteristics. In this group, there is a lack of evidence regarding excess weight and dyslipidemia in children, despite previous studies reporting important prevalences of obesity-related indicators, diabetes, and hypertension in Seri adults [13,14]. The Seri community is not genetically related to other indigenous groups in Mexico and is traditionally dedicated to fishing rather than agriculture [13].

Although commonly known as the Seri people, this name originates from the Yaqui community and translates as “men of the sand”, while their self-designation is Comcáac, meaning “the people” in their native language, Cmique Iitom [15]. Until the 20th century, they maintained a nomadic lifestyle based on fishing, hunting, and gathering; however, their territory is now restricted to Tiburón Island and a small section of the Sonoran coast, representing less than 10% of its original extent [13,15]. As part of a transition to a sedentary lifestyle, the population settled in two villages, Punta Chueca and El Desemboque. Despite these changes, they preserve ancestral traditions such as the making of coritas and fishing, which remains their principal economic activity [14].

For all the reasons stated above, this study aims to evaluate: 1. the prevalence of excess weight (including risk of overweight, overweight, and obesity) and dyslipidemia, and 2. the factors associated with BMI-for-age Z-score and dyslipidemia in indigenous Seri children.

## 2. Materials and Methods

This cross-sectional study was conducted in the two Seri villages and aimed to include all Seri children aged from 3 to 11 years residing in these communities at the time of data collection. Data were collected in Punta Chueca in November 2023 and in El Desemboque in September 2024.

Before data collection, permission was granted by the Elders Council and the Governor in both Punta Chueca and El Desemboque. Principals from preschools and elementary schools also authorized and supported the study. All participants, including children and their parents, provided assent or informed consent, respectively. The protocol for this study was evaluated and approved by the Research Ethics Committee of the Research Center for Food and Development, A.C. (registration CONBIOÉTICA-26-CEI-001-20200122), which operates in accordance with the Regulations of the General Health Law on Health Research of Mexico (protocol code CEI/028-1/2022, approved on 3 February 2023). The study was conducted in accordance with the principles of the Declaration of Helsinki.

### 2.1. Subjects

The prevalence of excess weight was assessed in 32 preschool (3–4 years) and 122 school-age (5–11 years) children, whereas the prevalence of dyslipidemia was assessed only in school-age children. Meanwhile, the factors associated with BMI-for-age Z-score and dyslipidemia were assessed among school-age children. Participants were recruited using attendance lists from local preschools and primary schools, provided by the school principals. To include all Seri children from both villages, an additional strategy was implemented to recruit those who were not enrolled in school at the time of the study, with the assistance of teachers and other community members.

Eligibility criteria for children included being aged 3 to 11 years at the time of recruitment, having at least one parent or grandparent with Seri ancestry [16], and agreeing to participate by signing the assent form. Mothers were eligible for the analysis if they were the biological mother of the participating child and had signed the informed consent form. Children and their mothers were excluded if they had a health condition or disease that prevented them from participating, or if they declined to participate.

Data collection was conducted at two different moments. Anthropometric measurements, blood pressure, and blood samples were obtained early in the morning after overnight fasting. Later the same day or on subsequent days, structured interviews were conducted at the participant’s home to collect information about household and lifestyle variables. All measurements and questionnaires were administered by trained health professionals.

### 2.2. BMI-for-Age Z-Score

Body weight and height were measured following standardized procedures using a digital scale (FG-150KBM, A&D Co., Ltd.; Tokyo, Japan) and a stadiometer (Holtain, Harpenden Portable Stadiometer, model 603VR, Holtain Ltd., Crymych; Wales, UK), respectively. Measurements were taken in duplicate with children barefoot and wearing light clothing; discrepant values were measured a third time. Data were entered into the WHO AnthroPlus software (version 1.0.4, World Health Organization; Geneva, Switzerland) to calculate BMI-for-age Z-scores.

The BMI-for-age Z-score indicator was used to estimate the prevalence of excess weight. In preschool children, Z-scores >1 and ≤2 were classified as a risk of overweight, >2 and ≤3 as overweight, and >3 as obesity. Among school-age children, Z-scores >1 and ≤2 were classified as overweight, and scores >2 as obesity [17].

### 2.3. Dyslipidemia

After a 10–12 h overnight fast, approximately 9 mL of blood samples was collected from school-age children via venipuncture. Samples intended for lipid profile analysis were drawn into serum separator tubes, while those for plasma glucose measurement were collected in tubes containing sodium fluoride and potassium oxalate as anticoagulants. Blood samples were centrifuged at 3400 rpm for 15 min to separate serum and plasma, which were then aliquoted into cryogenic vials and stored at −20 °C prior to transport to the CIAD laboratory. Upon arrival, samples were maintained at −70 °C until analysis [18]. All biochemical assays were performed in duplicate using standardized procedures and commercially available kits (Randox; Crumlin, UK). Children were classified as having dyslipidemia if they had any elevated serum levels of total cholesterol, triglycerides, low-density lipoprotein cholesterol, or low levels of high-density lipoprotein cholesterol. Elevated total cholesterol was defined as levels of 200 mg/dL or higher. For triglycerides, the cut-off point was 100 mg/dL or higher in children aged 5–9 years, and 130 mg/dL or higher in those aged 10–11 years. Elevated low-density lipoprotein cholesterol was defined as levels of 130 mg/dL or higher. A low high-density lipoprotein cholesterol level was defined as below 40 mg/dL [11].

### 2.4. Potential Associated Factors

General data were collected from children and their mothers. Full name, date of birth, sex, and school grade were collected from children; for mothers, only full name and date of birth were collected. Mothers’ weights and heights were measured, using the methodology of the International Society for the Advancement of Kinanthropometry [19], and their BMIs were calculated as their weight (kg) divided by their height squared (m^2^). Maternal nutritional status was classified according to WHO criteria as underweight (BMI < 18.5 kg/m^2^), normal weight (18.5 to <25 kg/m^2^), overweight (25 to <30 kg/m^2^), or obesity (≥30 kg/m^2^). Waist circumference was also measured, and mothers with a waist circumference ≥ 80 cm were classified as having abdominal obesity [20].

A brief version of the Initial History Questionnaire from the American Academy of Pediatrics was used to collect children’s personal health information, including birth weight, type of breastfeeding at birth, and family history of diabetes [21]. Children’s physical activity levels and the time they spent in sedentary behaviors (screen time) were collected using an adapted version of the Assessment of Physical Activity Levels Questionnaire [22]. Sleep patterns and sleeping time per night were evaluated using the Children’s Sleep Habits Questionnaire [23].

Information on children’s diet, including daily intake of energy and nutrients, sweetened beverages, and ultra-processed products, was assessed using a food frequency questionnaire (FFQ) specifically developed for this population, based on previously established FFQ development methodologies [24,25]. This instrument has not yet been formally validated or assessed for reproducibility; detailed information on its development is provided in the Supplementary Materials. The FFQ evaluated the frequency and quantity (in grams per day) of food and beverages consumed by the children in the 7 days preceding the interview. Estimates of energy and nutrient intake were obtained using Food Processor software version 11 (Trustwell™, Oak Brook, IL, USA).

Information about children’s living conditions at home was also collected. A socioeconomic assessment was conducted to gather key data, including parents’ education levels, the number of remunerative economic activities for each parent, and the availability of household services and electronic devices, which were used to calculate the Household Modernity Index [26]. Information on social programs supporting any family member living in the same household as the children was also obtained through a specific questionnaire [27]. The Latin American and Caribbean Food Security Scale was used to assess the level of food insecurity (FI) in the home, classifying households with food security (0 points), low FI (1–5 points), moderate FI (6–10 points), or severe FI (11–15 points) [27]. Finally, the Household Water Insecurity Experiences Scale was applied to determine the level of water insecurity in the home [28].

Additionally, the cardiovascular health (CVH) score was estimated as another possible factor associated with BMI-for-age Z-score and dyslipidemia. According to the American Heart Association’s Life’s Essential 8 framework, this indicator comprises eight components related to children’s health factors and behaviors. For analysis purposes, the BMI-for-age percentile component was excluded from the CVH score calculation to avoid overlapping with the BMI-for-age Z-score. The modified CVH score was calculated as the average of the remaining components, each scored from 0 to 100, with higher scores indicating a better CVH. Each component is described below. The Mediterranean Dietary Pattern for Americans III (MEPA-III) was assessed by matching FFQ data to the MEPA-III food groups, assigning ≥9 food groups the highest score and ≤2 the lowest. Non–HDL cholesterol was measured in serum samples, with values < 100 mg/dL considered optimal. Fasting plasma glucose was used to assess blood glucose, with levels < 100 mg/dL classified as optimal. Blood pressure status was determined from systolic and diastolic measurements with an automated monitor (Omron HEM-907XL; Kyoto, Japan) adjusted for age, sex, and height, and then categorized as optimal, elevated, or hypertension stage 1, 2, or 3 according to the American Academy of Pediatrics Guidelines. Time spent in moderate- or vigorous-intensity physical activity was collected and reported in minutes per week. Nicotine exposure was classified based on the tobacco use of any person living in the children’s house. Sleep time was estimated as the average number of hours the child slept per night, as reported using the Children’s Sleep Habits Questionnaire [29,30].

### 2.5. Data Management

Data were collected using two complementary methods. Printed forms were used during the measurement session to compile general participant information, anthropometric and blood pressure measurements, and blood sample collection. As part of quality control, checklists were used to verify children’s fasting status, ensure the collection of two blood samples (one for glucose and one for lipid analyses), and confirm completion of all required printed forms.

Data on children’s diet, physical activity, and sleep habits, as well as information on parents’ economic activities, household characteristics, and family participation in social support programs, were collected via digital PDF forms during interviews conducted in participants’ homes.

Following data collection, information from printed forms was entered into Excel spreadsheets (Microsoft, Redmond, WA, USA), while data from digital forms was extracted using Wondershare PDFelement Software (version 9.0.9, Wondershare Technology Co., Ltd.; Shenzhen, China). Data quality was ensured through a double-verification process conducted in pairs, followed by a group review. Dietary data were analyzed and cleaned using established procedures, as detailed elsewhere [31]. For the remaining variables, observations exceeding ±3 standard deviations from the mean were excluded [32], resulting in the removal of 2 observations for total screen time and 3 for weekly moderate-to-vigorous physical activity.

To minimize potential sources of bias, specific strategies were implemented. Selection bias was reduced by including all eligible Seri children from both villages, as well as those not enrolled in school; measurement bias was minimized through standardized data collection procedures and trained personnel; and information bias was addressed using structured and validated questionnaires.

### 2.6. Statistical Analysis

To describe participant characteristics, continuous variables are summarized using means and standard deviations, and categorical variables are presented as frequencies and percentages. Prevalences were reported as totals and compared between boys and girls; sex differences were evaluated using ordinal logistic regression.

Regarding missing data, anthropometric measurements were not obtained for one mother because she declined participation. Additionally, due to insufficient blood volume, lipid profiles could not be fully determined in 20 children. All analyses were performed using complete-case data for each outcome.

Variables associated with the BMI-for-age Z-score, treated as continuous, were analyzed using multiple linear regression. Variables associated with dyslipidemia were assessed using multiple logistic regression, given the dichotomous nature of the outcome. The final logistic regression model included 81 participants with complete data, of whom 39 were classified as dyslipidemia cases.

Variables considered as potential confounders were included in both regression analyses. Potentially associated variables were first evaluated with univariate analyses by the compliance of both of the following criteria: (1) biological plausibility of the measures of association (β or OR) based on prior evidence from studies in similar populations, and (2) obtaining a *p*-value ≤ 0.20. Variables that did not meet this criterion were excluded from the analysis. This was followed by stepwise selection (*p* ≤ 0.05) and preserving the criterion of biological plausibility of the measures of association for the variables included in the generated models. Effect modification by key variables such as sex and age (*p* ≤ 0.10), collinearity (variance inflation factor ≥ 10), and model assumptions were assessed for all final models. All analyses were conducted using Stata version 18, with a significance level of *p* ≤ 0.05. Sensitivity analyses were conducted by replacing indicators of maternal adiposity (weight, BMI, and waist circumference) and by modeling food insecurity in both dichotomous and continuous forms to ensure the consistency of the observed associations.

## 3. Results

**Figure 1:**
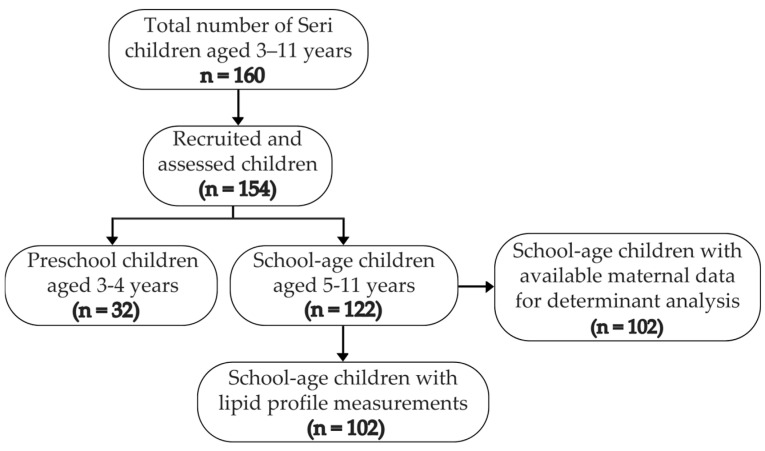
Flow diagram of participation and analytical subsets in Seri children.

According to the most recent official report, in 2020, the Seri community comprised 1011 individuals [33]. At the time of this study, 160 children aged 3 to 11 years resided in Punta Chueca or El Desemboque (Figure 1). Of these, 153 children were recruited from school attendance lists, and with the assistance of the school principal, one additional child not enrolled in school was identified at home. Six children declined participation, resulting in a participation rate of 96%.

Table 1 presents the anthropometric, cardiovascular, and lifestyle characteristics of Seri children, showing an equal sex distribution in the total sample (50.0% girls and 50.0% boys). The mean CVH score was 83.1, with higher scores in preschool than in older children. Most participants were classified as sedentary, with a higher prevalence among preschoolers (71.9%) than among school-aged children (65.6%). Macronutrient distribution was similar across both age groups; however, school-aged children reported higher intakes of dietary fiber and cholesterol.

**Figure 2:**
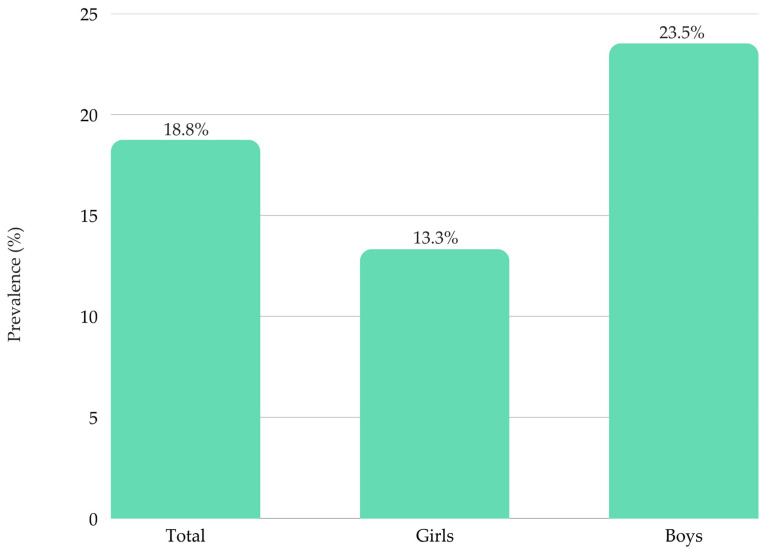
Prevalence of risk of overweight in preschool Seri children (n = 32).

**Figure 3:**
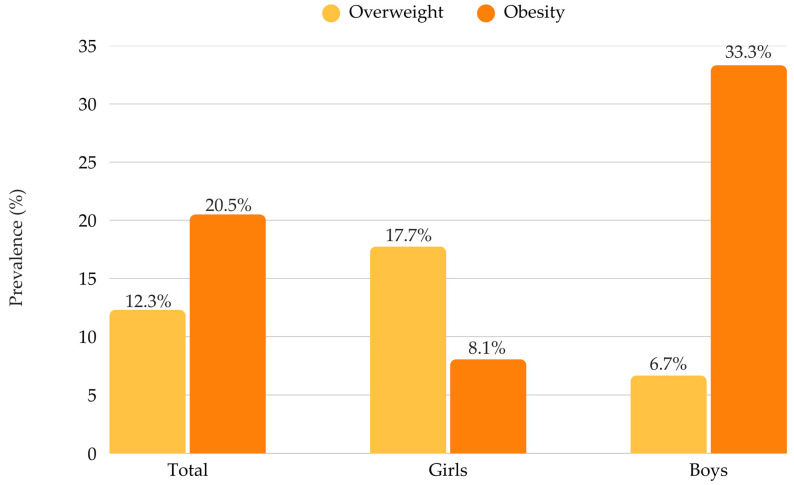
Prevalence of overweight and obesity among school-age Seri children (n = 122).

The resulting prevalences of excess weight by age group are shown in Figure 2 and Figure 3. Among preschoolers (Figure 2), no cases of overweight or obesity were identified, although 18.8% showed a risk of overweight (girls: 13.3%; boys: 23.5%). In school-age children (Figure 3), obesity was the most prevalent condition (20.5%), occurring in 33.3% of boys and 8.1% of girls, whereas overweight was observed in 17.7% of girls and 6.7% of boys. Overall, boys were more likely to belong to a higher excess weight category than girls (OR = 2.15, *p* = 0.042).

**Figure 4:**
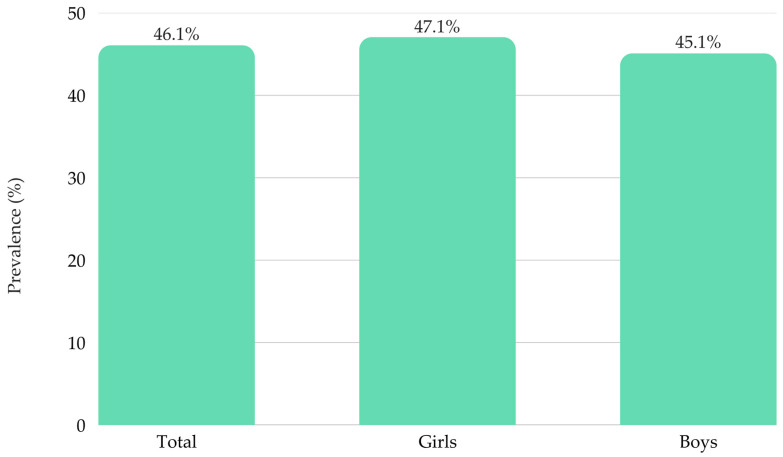
Prevalence of dyslipidemia among school-age Seri children (n = 102).

During blood sample processing, lipid profile measurements were not obtained in 20 participants due to insufficient sample volume; therefore, the analysis includes lipid indicators from 102 school-age children. Overall, 46.1% of the children presented dyslipidemia, with similar prevalences between girls (47.1%) and boys (45.1%) (Figure 4). Regarding dyslipidemia components (Table 2), low levels of high-density lipoprotein cholesterol were the most prevalent (hypoalphalipoproteinemia, 35.3%).

To analyze factors associated with BMI-for-age Z-score in school-age Seri children, a multiple linear regression was performed with this indicator as the dependent variable. Maternal obesity indicators were considered a potential factor associated; however, because the analysis was restricted to biological mothers, the models included only 102 children with this data. Two models were identified after a sensitivity analysis using maternal obesity indicators (Table 3). In Model 1, a higher maternal weight (β = 0.02, *p* = 0.001) and being a boy (β = 0.62, *p* = 0.008) were positively associated with BMI-for-age Z-score, whereas a greater FI (β = −1.96, *p* = 0.050) and higher CVH score (β = −0.07, *p* < 0.001) were negatively associated. In Model 2, higher maternal BMI (β = 0.07, *p* = 0.001) and a higher modernity index (β = 0.15, *p* = 0.013) showed positive associations, while a greater FI (β = −1.33, *p* = 0.002) and longer sleep duration (β = −0.36, *p* < 0.001) showed negative associations.

Multiple logistic regression analysis was conducted with dyslipidemia as the dependent variable, and a single model was identified (Table 4). Maternal obesity indicators were also included as potential associated factors, which restricted the analysis to children with available maternal data; additionally, lipid measurements were unavailable for 20 children due to insufficient blood sample volume. Given the above, the final sample size for this analysis was 81 subjects. Maternal overweight (OR = 4.43, *p* = 0.038), a higher percentage of energy intake from ultra-processed products (OR = 1.04, *p* = 0.036), having a father working as a merchant (OR = 6.99, *p* = 0.017), and an older age (OR = 1.44, *p* = 0.027) were positively associated with dyslipidemia. Maternal obesity was not significantly associated with the outcome (OR = 0.76, *p* = 0.694). In contrast, a higher number of remunerative economic activities performed by the mother (OR = 0.45, *p* = 0.022) was negatively associated with dyslipidemia.

## 4. Discussion

This study evaluated the prevalence and associated factors of excess weight and dyslipidemia among Seri indigenous children of Sonora. In summary, excess weight is common in both age groups and dyslipidemia among school-aged children. Additionally, several maternal, household, and individual-level factors were associated with these two health conditions.

Regarding the risk of overweight in preschool Seri children, a prevalence of 18.8% was observed. The most recent national prevalence estimates from the 2012 National Health and Nutrition Examination Survey found that 23.8% of Mexican preschoolers were at risk of overweight [34]. These findings suggest that the risk of overweight emerges early in Mexican children, underscoring the importance of early monitoring, as this condition represents an initial stage of excess weight [17]. Notably, no preschool Seri children were classified as overweight or obese, in contrast to the national (7.5%) and state-level (10.1%) prevalence of excess weight reported for this age group [8].

A similar pattern was observed among school-age Seri children, although with important differences. The combined prevalence of overweight and obesity (32.8%) was slightly lower than the national prevalence among indigenous (36.4%) and non-indigenous children (36.6%). At a national level, overweight is more prevalent than obesity in this age group. A similar pattern has been reported among Yaqui school-age children, with a higher prevalence of overweight than obesity [9]. In contrast, among Seri children, obesity accounts for the largest proportion of excess weight. This evidence highlights heterogeneity in excess weight patterns across indigenous communities and, by comparison, with national prevalence rates.

The present study found a total prevalence of dyslipidemia of 46.1% among school-age Seri children. This result is consistent with previous studies in Mexican indigenous populations, which reported prevalences of 46.3% among Seri children from Punta Chueca and 38.6% among school-age children from two Yaqui villages [11,12]. These high rates are concerning, given that dyslipidemia in childhood is frequently accompanied by insulin resistance, non-alcoholic fatty liver, and an increased risk of atherosclerotic cardiovascular disease [35].

Overweight and obesity are risk factors for metabolic alterations such as dyslipidemia. In the analysis of school-age Seri children, 44.7% of those with dyslipidemia were also classified as overweight or obese (Table 2). This finding underscores the importance of assessing lipid alterations from early life, particularly in populations at high cardiometabolic risk, and highlights the need to evaluate dyslipidemia even in childhood, especially among children with excess weight [4].

These findings suggest that variables at multiple levels, including maternal, household, and individual characteristics, contribute to excess weight in this population. Factors associated with BMI-for-age Z-score have been identified in Mexican school-age children. The most frequently reported include greater time spent in sedentary activities, maternal obesity, and higher maternal education level, all of which are positively associated with excess weight, whereas a higher FI score has been negatively associated [9,36,37].

Maternal nutritional status was associated with the BMI of Seri children, as shown in both models (models 1 and 2). Given the documented influence of parental eating behaviors on children’s dietary patterns [38], the transition of Seri adults toward less healthy eating habits may explain this finding [14,33].

Similarly, two household-related variables, FI and the modernity index, were also found to be associated with the BMI-for-age Z-score of Seri school-age children. FI reflects household food-related experiences, whereas the modernity index indicates the level of household assets. FI was inversely associated with BMI-for-age Z-score in Seri children in both Models 1 and 2. The relationship between FI and excess weight is complex and remains controversial, with evidence suggesting a positive association in high-income populations but an inverse pattern in low and middle-income countries [25]. In line with these findings, our results are consistent with previous reports in Mexican children [9,36]. In the Seri population, FI may reflect limited economic access and, consequently, dietary patterns more closely aligned with locally available traditional foods, such as seafood. Nevertheless, dietary patterns according to FI status were not directly evaluated in the present analysis, and alternative explanations cannot be ruled out.

In contrast to FI, the modernity index, another household-related variable, was positively associated with the BMI-for-age Z-score among Seri children. Although this factor has not been previously evaluated in children, it may be comparable to the well-being index, as both are based on household characteristics and assets. Consistent with these findings, a 2020 national analysis found that a higher well-being index was a positive predictor of obesity in Mexican children [37]. Additionally, a study of Yaqui adults found that the modernity index is associated with BMI and a lifestyle-related risk factor.

In addition, three individual-level health-related variables were identified as factors associated with BMI-for-age Z-score among Seri school-age children: the CVH score, sleep duration assessed as the average number of hours slept per night, and sex.

The CVH score reflects children’s lifestyle and has been associated with health outcomes in other populations; higher scores indicate better cardiovascular health, on a scale ranging from 0 to 100 [39]. There is no evidence that previous studies have evaluated this indicator in the Mexican population, and internationally, it has not been examined in relation to indicators associated with excess weight in children. In this context, in Model 1, the CVH score was inversely associated with overweight and obesity, a finding that may be explained by its composite nature, which captures multiple dimensions relevant to excess weight. Importantly, all eight components of the CVH score are modifiable indicators of individual health, underscoring their potential relevance for early prevention strategies.

Sleep duration also emerged as a relevant associated factor in Model 2. Previous research in Mexican children and adolescents has shown that short sleep duration increases the risk of overweight and obesity by approximately 16% [40]. Aligned with these findings, among Seri school-age children, each additional hour of sleep was associated with a 0.36-unit decrease in the BMI-for-age Z-score.

Regarding sex, being a boy was associated with a higher BMI-for-age Z-score in the present study. By comparison, previous studies in Mexican children reported that girls have a lower risk of excess weight [7]. These findings suggest sex-related differences in growth patterns and other individual health characteristics.

After multiple logistic regression analyses, a single model was identified that included the variables associated with dyslipidemia in school-age Seri children. However, the dyslipidemia model should be interpreted as exploratory due to the limited sample size.

Maternal BMI, an indicator of maternal nutritional status, was included in the dyslipidemia model, with a positive association. This finding is novel, as maternal nutritional status has not been previously reported as a factor associated with dyslipidemia in children.

Among dietary variables, the percentage of energy intake from ultra-processed products was positively associated with dyslipidemia in Seri children. Such products have been associated with adverse lipid profiles in children from other populations, including higher levels of total cholesterol, triglycerides, and low-density lipoprotein cholesterol, as well as lower high-density lipoprotein cholesterol [41]. This result suggests that consumption of ultra-processed foods may be associated with dyslipidemia in this population, although prospective studies are needed to confirm this relationship.

Moreover, two indicators related to parental economic activities were associated with dyslipidemia in Seri children. The maternal remunerative activities variable refers to the total paid economic activities in which the mother engages, while the father merchant variable indicates whether the father is engaged in these activities. A higher number of maternal remunerative activities was inversely associated with dyslipidemia, whereas paternal involvement in commerce was linked to an increased risk. However, the association with the father merchant variable should be interpreted with caution, as the wide confidence interval suggests limited precision, likely due to the small number of participants in this subgroup. Rather than reflecting household income alone, these indicators may capture broader household dynamics affecting food acquisition, availability, and consumption. Comparable associations have been reported in other child populations, where paternal labor status and extended parental working hours have been identified as factors associated with dyslipidemia [11,42].

Finally, children’s age was another individual-level health-related variable associated with dyslipidemia. This finding is consistent with evidence that blood lipids change throughout childhood, given the physiological changes of development [4].

As described in the Materials and Methods section, in preschool children, only the prevalence of excess weight was evaluated, along with a description of their characteristics. Although 91% of Seri preschool children were included, the sample size (n = 32) in this age group was too small to analyze factors associated with BMI-for-age Z-score. Moreover, analyses of associated factors are typically conducted separately by age group in the literature, and combining preschool and school-age children would still not be feasible, given that preschoolers account for only a quarter of the school-age sample.

The present study has several limitations that should be considered. Although dietary intake was assessed using an FFQ specifically developed for the Seri population, this instrument has not yet been formally validated or assessed for reproducibility; therefore, estimates of energy and nutrient intake, as well as sweetened beverage and ultra-processed food consumption, should be interpreted with caution. Future studies should evaluate the validity and reproducibility of this instrument in Seri children. Furthermore, due to its cross-sectional design, it is not possible to establish temporal relationships between the identified variables and excess weight or dyslipidemia. However, the reduced number of subjects may have contributed to the limited precision of some estimated associations; therefore, these findings should be interpreted with caution.

Despite this, our findings are consistent with evidence from cohort studies reporting associations between excess weight and children’s sleep duration [43], maternal health indicators [44,45], and family socioeconomic conditions [46]. In contrast, evidence on factors associated with dyslipidemia in children remains limited, and most available studies have been conducted in adult populations, where comparable associations have been observed [47,48]. In addition, lipid measurements were not available for a proportion of school-age children, which may have introduced selection bias. This is important because if children without lipid measurements differed from those included in the analyses, the observed associations may have been under- or overestimated.

Nevertheless, this proportion was relatively small, and the study still provides relevant evidence on blood lipids and dyslipidemia in this population, where such information remains scarce.

A key strength of this study is the inclusion of nearly all Seri children within the study age range, along with a high response rate (96%), which enhances the representativeness of the population and supports the validity of prevalence estimates.

Taken together, these findings indicate that overweight, obesity, and dyslipidemia are present in Seri children and are determined by a combination of maternal nutritional status, household economic dynamics, lifestyle behaviors, and dietary patterns. This multifactorial context highlights the complexity of these health conditions and underscores the need to consider multiple factors associated with BMI-for-age Z-score and dyslipidemia in Seri children.

## 5. Conclusions

In summary, this study provides new evidence on alterations in nutritional and health status, identifying a high prevalence of risk of overweight among preschool Seri children and of overweight, obesity, and dyslipidemia among school-age Seri children. These findings are especially relevant in preschoolers, an understudied age group. Excess weight and dyslipidemia were associated with maternal nutritional status, household-related, and individual-level health variables; additionally, dyslipidemia was associated with a dietary-related variable. The present study highlights the multifactorial nature of excess weight and lipid alterations in indigenous children and emphasizes the importance of considering their specific cultural environment and needs. Overall, this work provides contextualized evidence on the prevalence of excess weight and dyslipidemia and their associated factors in indigenous children in Mexico, which may serve as a basis for future research and comparative analyses in Seri and similar populations.

## Figures and Tables

**Table 1:** Summary of the anthropometric, cardiovascular, and lifestyle characteristics of Seri children by age group.

Variable	Total (n = 154)	Preschoolers (n = 32)	School-Age (n = 122)
Sex			
girls	77 (50.00)	15 (46.88)	62 (50.82)
boys	77 (50.00)	17 (53.13)	60 (49.18)
Weight (kg)	28.91 ± 12.14	17.12 ± 2.25	32.01 ± 11.77
Height (cm)	126.55 ± 16.24	104.92 ± 5.25	132.22 ± 13.04
Physical activity level			
sedentary	103 (66.88)	23 (71.88)	80 (65.57)
moderately active	44 (28.57)	9 (28.13)	35 (28.69)
very active	7 (4.55)	0 (0)	7 (5.74)
Cardiovascular health score ^1^	83.11 ± 10.27	85.86 ± 8.73	82.39 ± 10.55
MEPA-III score ^2^	75.78 ± 21.01	80.63 ± 14.35	74.51 ± 22.30
Time of mod-vig activities (min/week)	7.69 ± 11.68	8.29 ± 15.91	7.54 ± 10.47
BMI-for-age (percentile)	57.16 ± 31.95	52.71 ± 28.20	58.33 ± 32.87
Fasting plasma glucose (mg/dL)			91.67 ± 8.45
Non-HDL cholesterol (mg/dL) ^3^			95.88 ± 24.81
Systolic blood pressure (mmHg)	92.96 ± 9.36	88.43 ± 8.39	94.01 ± 9.29
Diastolic blood pressure (mmHg)	54.58 ± 7.38	52.79 ± 5.19	55.00 ± 7.76
Sleeping time (h/night)	9.60 ± 1.40	10.15 ± 1.11	9.45 ± 1.43
Nicotine exposure			
not exposed	109 (74.15)	22 (75.86)	87 (73.73)
exposed	38 (25.85)	7 (24.14)	31 (26.27)
Energy intake (kcal/day)	1796.78 ± 839.98	1191.63 ± 574.31	1953.12 ± 828.36
Energy from carbohydrates (%)	55.79 ± 6.27	55.18 ± 6.85	55.94 ± 6.13
Energy from proteins (%)	11.56 ± 2.29	11.89 ± 2.52	11.47 ± 2.22
Energy from fats (%)	32.66 ± 5.03	32.92 ± 5.57	32.59 ± 4.90
Dietary cholesterol (mg/day)	275.16 ± 186.36	214.43 ± 149.74	291.09 ± 192.20
Dietary fiber (g/day)	18.81 ± 9.43	12.73 ± 7.13	20.41 ± 9.33
Energy from ultra-processed (%) ^4^	40.24 ± 14.56	36.99 ± 15.17	41.09 ± 14.33
Sedentary activity time (h/day)	3.53 ± 3.31	2.88 ± 2.86	3.69 ± 3.42

Mod-vig: physical activity with moderate to vigorous intensity. ^1^ The cardiovascular health score represents the average of eight health components, following the methodology of the American Heart Association’s “Life’s Essential 8”. ^2^ Scoring for diet quality according to the Mediterranean Dietary Pattern for Americans (MEPA-III). ^3^ Calculated in 102 children due to insufficient blood samples in the remaining participants. ^4^ Of the total daily energy intake, this is the percentage derived from ultra-processed products. Quantitative variables are expressed as mean ± standard deviation; categorical variables as frequency and percentage n (%).

**Table 2:** Prevalence of dyslipidemia and lipid components in Seri school-age children (n = 102).

Variable	Girls	Boys	Total
	f (%)	f (%)	f (%)
Dyslipidemia ^1^	24 (47.06)	23 (45.10)	47 (46.08)
Dyslipidemia + Excess weight *	12 (50.00)	9 (39.13)	21 (44.68)
Hypoalphalipoproteinemia ^2^	19 (37.25)	17 (33.33)	36 (35.29)
Hypertriglyceridemia ^3^	4 (7.69)	4 (7.41)	8 (7.55)
Hyperlipoproteinemia ^4^	2 (3.92)	3 (5.88)	5 (4.90)
Hypercholesterolemia ^5^	1 (1.92)	2 (3.70)	3 (2.83)

Data are presented as frequency (percentage). ^1^ Dyslipidemia was defined as the presence of at least one high or low lipid parameter, including elevated serum total cholesterol, triglycerides, or low-density lipoprotein cholesterol (LDL cholesterol), or reduced high-density lipoprotein cholesterol (HDL cholesterol). * Children with dyslipidemia who were also classified as overweight or obese. ^2^ Hypoalphalipoproteinemia was defined as HDL cholesterol < 40 mg/dL. ^3^ Hypertriglyceridemia was classified by age: triglycerides ≥ 100 mg/dL in children younger than 10 years and ≥130 mg/dL in those aged 10 years or older. ^4^ Hyperlipoproteinemia was defined as LDL cholesterol ≥ 130 mg/dL. ^5^ Hypercholesterolemia was defined as total cholesterol ≥ 200 mg/dL.

**Table 3:** Factors associated with BMI-for-age Z-score in Seri school-age children (n = 102).

**Model 1**			
**Variable**	**β**	***p*** **Value**	**CI 95%**
Maternal weight (kg)	0.02	0.004	0.008–0.040
Food insecurity (1: yes)	−1.33	0.002	−2.149–−0.513
Cardiovascular health score	−0.03	0.018	−0.060–−0.006
Sex (1: boys)	0.73	0.008	0.198–1.260
**Model 2**			
**Variable**	**β**	***p*** **value**	**CI 95%**
Maternal BMI (kg/m^2^)	0.07	0.001	0.030–0.115
Food insecurity (1: yes)	−1.33	0.002	−2.161–−0.507
Modernity index	0.15	0.013	0.032–0.266
Sleep time (h/night)	−0.36	<0.001	−0.549–−0.162

BMI: Body mass index. CI: Confidence interval. Values reported as *p* = 0.000 were expressed as *p* < 0.001.

**Table 4:** Factors associated with Dyslipidemia in Seri school-age children (n = 81).

Variable	OR	*p* Value	CI 95%
Maternal BMI (1: under/normal weight)			
overweight	4.43	0.038	1.087–18.077
obesity	0.76	0.694	0.198–2.944
Energy from ultra-processed (%) *	1.04	0.036	1.003–1.080
Economic activities of the mother **	0.45	0.022	0.222–0.892
Father merchant (1: yes)	6.99	0.017	1.407–34.705
Age (years)	1.44	0.027	1.041–1.981

* Percentage of total daily energy intake derived from ultra-processed products. ** Number of paid economic activities the mother engages in (e.g., selling traditional Seri crafts, tourism activities). BMI: Body mass index. CI: Confidence interval.

## Data Availability

The data of this study are available from the corresponding author upon reasonable request.

## Supplementary Materials

The following supporting information can be downloaded at https://www.mdpi.com/article/10.3390/epidemiologia7030084/s1. Table S1: Grouping of items in the FFQ for Comcáac children.

## Abbreviations

The following abbreviations are used in this manuscript:

BMI	Body mass index
FFQ	Food frequency questionnaire
FI	Food insecurity
CVH	Cardiovascular health
MEPA-III	Mediterranean dietary pattern for Americans III
