# Supplementary material for: Excess Weight and Dyslipidemia in Seri (Comcáac) Indigenous Children: A Cross-Sectional Study of Prevalences and Associated Factors
Source: Epidemiologia (Basel). 2026 Jun 16;7(3):84. doi: 10.3390/epidemiologia7030084 (PMC13298066; doi:10.3390/epidemiologia7030084)
Supplement: Supplementary file 1 [file epidemiologia-07-00084-s001.zip › epidemiologia-4265398-supplementary.pdf]

Supplementary Material

Food Frequency Questionnaire Development

A culturally adapted Food Frequency Questionnaire (FFQ) was developed to assess habitual dietary intake among Seri (Comcáac) indigenous children aged 2–11 years from Punta Chueca and El Desemboque, Sonora, Mexico.

The FFQ was based on 24-hour dietary recalls (24DRs) collected between July and August 2023. A total of 68 children were initially recruited; 4 were excluded due to unreliable dietary information, and 5 additional 24DRs were excluded as outliers according to predefined criteria. The final analytic sample included 63 children.

Dietary data were collected by trained nutritionists through household visits using the standard 24DR methodology. Children were interviewed with the assistance of their mothers or primary caregivers. Food models and household measuring utensils (cups, spoons, plates, and glasses) were used to improve portion size estimation. Traditional Seri foods and commonly consumed commercial products were incorporated into the food models used for portion estimation.

The FFQ development followed established procedures used in Mexican population-based dietary surveys and previous FFQ development studies in Indigenous populations from northwestern Mexico. Information obtained from the 24DRs was used to identify commonly consumed foods, estimate portion sizes, and design the FFQ structure.

Food selection process

A total of 181 foods and beverages were identified from the 24DRs. The percentage contribution (PC) of each item to total energy and nutrient intake was calculated. Items were ranked according to their PC to energy, carbohydrates, proteins, lipids, cholesterol, and fiber intake. Foods contributing to the top 90% of intake for each nutrient were selected to form the preliminary food list. After merging nutrient-specific lists and removing duplicates, 80 items were retained.

A focus group with 25 members of the Seri community (children and adults) was conducted to assess cultural relevance and completeness of the FFQ. Participants reviewed all items and suggested additional foods commonly consumed by children. Eight foods were added based on this process: fresh scallops, cucumber, grilled fish, coffee with sugar and/or milk, lentil soup, chicken broth, fish broth, and bacon. The final FFQ included 88 food and beverage items grouped into 12 categories.

Portion size estimation

Portion sizes were estimated using the median intake reported in the 24DRs. Medium portions corresponded to the median of consumption; small and large portions were defined as 0.5 and 1.5 times the median intake, respectively.

Because preschool-aged children generally consume smaller amounts, adjusted portion sizes were developed for children under 5 years. Two versions of the FFQ were created: one for preschool and one for school-aged children. Food items were identical in both versions, differing only in portion-size estimates.

Table S1. Grouping of items in the FFQ for Comcáac children

Groups	Items
Fruits	Cherries, apple, melon, banana, grapes
Vegetables	Zucchini, onion, green Anaheim pepper, lettuce, cucumber, tomato
Sweet and salty snacks	Amaranth bar, bolis*, muffins/pancakes, potato chips (any brand), industrialized cookies (any flavor), helado**, ice cream, birthday cake (any flavor), raspado***
Dairy	Whole milk, powdered milk, Nutri® Milk (ultra pasteurized dairy product with vegetable fat), processed cheese slices, asadero cheese (grated)
Seafood	Fresh scallops, fried scallops, roasted fish, fried fish
Beverages	Natural water, drinks with natural fruit and added sugar (i.e. lemonade), coffee, regular Coke® (or any soda flavor), industrialized juices, teas and nectars (any flavor), hot/cold cocoa
Miscellaneous	Guacamole Ψ, condensed milk, margarine, mayonnaise, half-and-half cream, refined sugar, vegetable oil, canned tomato sauce
Legumes	Refried Beans (canned or homemade), lentil soup
Prepared dishes	Fish broth, chicken broth, beef broth, menudo∞, pozole§, pasta (spaghetti, macaroni, etc.), Knorr® instant soup, breakfast cereals, Maruchan® instant soup, nachos with cheese, chicken nuggets/tenders, pizza
Cereals and potatoes	Rice (boiled or steamed rice cooked with or without oil), canned corn, whole wheat loaf bread, Seri bread±, Virginia bread¥, potato (stewed or in broth), French fries, totopos (tortilla chips)
Eggs and meats	Beef (grilled, steak, shredded, machaca£ or ground), eggs (fried, scrambled with ham, chorizo+, machaca, or sausage), ham, chicken (wings, thighs, breast, drumsticks), sausage (pork), bacon, grilled venison meat
Tortillas	Flour tortillas, corn tortillas

**Bolis in Sonora is a frozen snack that contains flavored juice; **Helado is a frozen dessert made with a mixture of milk, natural fruit, and/or chocolate; ***Raspado is similar to shaved ice or a snow cone, but is made with natural fruit syrup; Ψ Guacamole is mashed avocados, usually seasoned with salt, pepper, and lemon juice; ∞ Menudo is a Sonoran broth made with corn kernels and beef tripe; § Pozole is a traditional Mexican dish made with corn kernels cooked with pork or beef and seasoned with various spices; ± Pan Seri is a traditional bread from the Comcáac community; ¥ Virginia bread is a homemade Mexican bread, similar to sandwich bread, the name and preparation vary depending on the region; £ Machaca is a type of dried, shredded and seasoned beef, commonly used in Sonoran cuisine; + Chorizo is a cured sausage made from meat, Mexican chorizo is seasoned with chili, garlic, and spices.*
